# Infrared-pump electronic-probe of methylammonium lead iodide reveals electronically decoupled organic and inorganic sublattices

**DOI:** 10.1038/s41467-019-08363-2

**Published:** 2019-01-29

**Authors:** Peijun Guo, Arun Mannodi-Kanakkithodi, Jue Gong, Yi Xia, Constantinos C. Stoumpos, Duyen H. Cao, Benjamin T. Diroll, John B. Ketterson, Gary P. Wiederrecht, Tao Xu, Maria K. Y. Chan, Mercouri G. Kanatzidis, Richard D. Schaller

**Affiliations:** 1 Center for Nanoscale Materials, Argonne National Laboratory, 9700 South Cass Avenue, Lemont, IL 60439 USA; 2 Department of Chemistry and Biochemistry, Northern Illinois University, 1425 W. Lincoln Hwy., DeKalb, IL 60115 USA; 3 Department of Chemistry, Northwestern University, 2145 Sheridan Road, Evanston, IL 60208 USA; 4 Materials Science Division, Argonne National Laboratory, 9700 South Cass Avenue, Lemont, IL 60439 USA; 5 Department of Physics and Astronomy, Northwestern University, 2145 Sheridan Road, Evanston, IL 60208 USA

## Abstract

Organic-inorganic hybrid perovskites such as methylammonium lead iodide (CH_3_NH_3_PbI_3_) are game-changing semiconductors for solar cells and light-emitting devices owing to their defect tolerance and exceptionally long carrier lifetimes and diffusion lengths. Determining whether the dynamically disordered organic cations with large dipole moment benefit the optoelectronic properties of CH_3_NH_3_PbI_3_ has been an outstanding challenge. Herein, via transient absorption measurements employing an infrared pump pulse tuned to a methylammonium vibration, we observe slow, nanosecond-long thermal dissipation from the selectively excited organic mode to the inorganic sublattice. The resulting transient electronic signatures, during the period of thermal-nonequilibrium when the induced thermal motions are mostly concentrated on the organic sublattice, reveal that the induced atomic motions of the organic cations do not alter the absorption or the photoluminescence response of CH_3_NH_3_PbI_3_, beyond thermal effects. Our results suggest that the attractive optoelectronic properties of CH_3_NH_3_PbI_3_ mainly derive from the inorganic lead-halide framework.

## Abstract (web summary)

It has been challenging to probe whether dynamically disordered organic cations affect optical properties of CH3NH3PbI3. Here, Guo et al. employ infrared-pump electronic-probe spectroscopy and show that pump-induced atomic motions of the organic cations do not substantially alter optoelectronic properties.

## Introduction

Metal-halide perovskites are emerging as low-cost, solution-processable semiconductors for applications involving the harvesting and emitting of light^1,2^. Recent spectroscopic measurements have demonstrated that the favorable optical and electronic properties of these materials likely arise from the strong electron–phonon interactions in these soft semiconductors, in conjunction with the large dynamic disorder of the lead-halide octahedral framework^3–6^. Hybrid organic–inorganic metal-halide perovskites, including the prototypical CH_3_NH_3_PbI_3_, present additional challenges in understanding the interplay between the structural and optoelectronic properties. This primarily stems from the significant structural fluctuations of the organic cations (e.g., CH_3_NH_3_^+^) with picosecond relaxation times observed from neutron scattering and millmeter-wave spectroscopic experiments^7,8^. Due to the potentially crucial importance for enhancing device efficiency^9^ and enabling additional functionalities^10,11^, the mutual interactions between the fluctuating dipolar organic cations and the charge carriers that reside on the inorganic Pb-I framework have been extensively investigated. Formation of (anti)ferroelectric domains arising from the orientational ordering of the polar organic cations was proposed to enhance carrier lifetimes^12^, but spectroscopic measurements did not support such a picture^13–15^. Dynamic bulk Rashba effect induced by the CH_3_NH_3_^+^ cations was also suggested^16,17^, yet recent theoretical and experimental results show that such an effect is negligible^18^. In fact, HC(NH_2_)_2_PbI_3_-based devices exhibit conversion efficiencies comparable to the CH_3_NH_3_PbI_3_ analog^19^, albeit the dipole moment of HC(NH_2_)_2_^+^ is much smaller than that of CH_3_NH_3_^+^ (ref. ^12^), and approaches that of the non-polar Cs^+^ cation. Although spectroscopy-based comparative studies between hybrid and all-inorganic perovskites have been attempted so as to evaluate the role of polar organic cations on material performance^20–22^, the properties of metal-halide perovskites are known to depend on synthetic conditions and material history^23,24^. For example, high-quality, vapor-deposited CsPbI_3_ thin films present impressively long carrier lifetime exceeding 10 µs that is comparable to the best CH_3_NH_3_PbI_3_ counterparts, whereas solution-processed CsPbI_3_ film showed significantly shorter lifetime^25^. Remarkable solar cell efficiencies derived from carefully prepared CsPbI_3_ have been reported^26,27^, and solar cells made from CH_3_NH_3_PbBr_3_ and CsPbBr_3_ exhibited comparable efficiencies^21^. These device characteristics hinted that the main role played by the organic cations is structural, with a different size than Cs^+^ as well as the additional electrostatic interactions and hydrogen bonding with the lead-halide framework^28^. While the black phase of CH_3_NH_3_PbI_3_ is stable at room temperature, it is known that the CsPbI_3_ counterpart is not stable at room temperature and special treatments are required to stabilize its black phase^29^, which makes a fair comparison between CH_3_NH_3_PbI_3_ and CsPbI_3_ difficult. As a result, experimental approaches permitting the direct probing of interactions between the organic and inorganic sublattices are important for the study of hybrid material systems^30^. Here, we employ infrared pump electronic-probe (IPEP) spectroscopy to excite the strongly absorbing vibrational modes of the organic sublattice and examine the corresponding optical response near the bandgap of CH_3_NH_3_PbI_3_. We found that the pump-induced motions of the CH_3_NH_3_^+^ cations do not change the subsequent absorption or the photoluminescence response of CH_3_NH_3_PbI_3_, but merely result in the heating of the inorganic sublattice in hundreds of picoseconds to nanosecond timescale.

## Results

### Static optical response

**Fig. 1:**
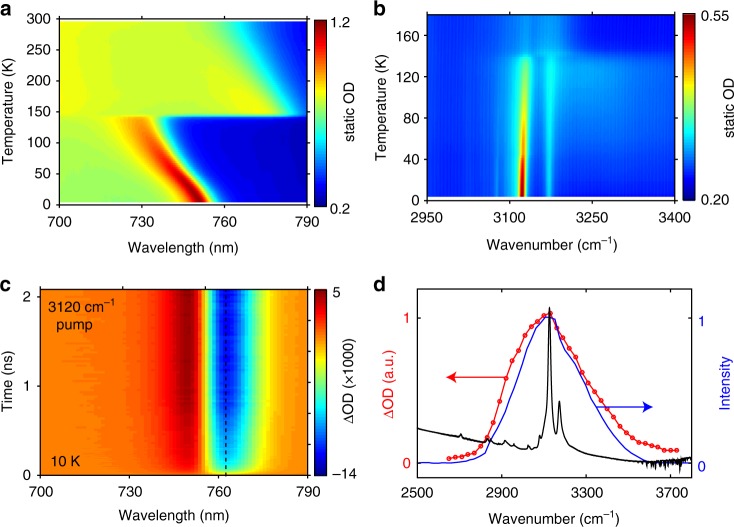
Static and infrared pump electronic-probe (IPEP) measurements of CH_3_NH_3_PbI_3_. **a** Static, temperature-dependent absorbance around the bandgap. **b** Static, temperature-dependent absorbance around the N-H-*as* vibrational modes. **c** ΔOD spectral map measured with on-resonance infrared pump (centered at 3120 cm^−1^) at 10 K. The dashed line shows the wavelength at which the kinetics are extracted (Fig. 2b). **d** Red: dependence of the magnitude of the strongest bleach signal on the center wavenumber of the pump pulse with a fixed fluence (measured at 10 K). Blue: spectral profile of the pump pulse centered at 3120 cm^−1^. Black: static absorbance of CH_3_NH_3_PbI_3_ (in arbitrary units) at 80 K

Figure 1a maps the temperature-dependent static absorbance of a CH_3_NH_3_PbI_3_ thin film around its bandgap. The tetragonal-to-orthorhombic phase transition near 145 K is denoted by an abrupt change of the bandgap, and for each individual phase the bandgap increases with temperature. The near-bandgap, distinct absorption peak in the orthorhombic phase arises due to a comparatively stronger excitonic character. Figure 1b presents the temperature-dependent static absorbance arising from the N-H asymmetric stretching modes (abbreviated here as N-H-*as* modes) of the CH_3_NH_3_^+^ cation, which exhibit the strongest optical absorption cross-section among various vibrational modes of the molecular cation^31^. A dramatic increase of the N-H-*as* absorbance accompanying the phase transition is due to the formation of hydrogen bonding that suppresses the rotational motions of CH_3_NH_3_^+^ in the orthorhombic phase^32^. The pronounced mid-infrared absorbance, in conjunction with the near-bandgap optical response, facilitate IPEP measurements on the orthorhombic phase of CH_3_NH_3_PbI_3_.

### IPEP measurements

In IPEP measurements, we selectively pumped the N-H-*as* modes (centered at 3120 cm^−1^; Fig. 1d and Supplementary Figure 1 show the pump profile) and probed the change of optical density (ΔOD) near the bandgap. The ΔOD spectral map acquired on CH_3_NH_3_PbI_3_ at 10 K is displayed in Fig. 1c. We observe a derivative-like transient spectral response, whose magnitude following the excitation starts from zero, grows with pump–probe time delay, and saturates on the nanosecond timescale. Positive ΔOD signal on the blue side of the static absorption peak and negative signal to the red conveys a spectral blueshift, consistent with transient lattice heating (Fig. 1a). Note that large bandgap shifts arising from the optical Stark effect were observed at zero delay time, when the pump and probe pulses temporally overlapped^33^ (Supplementary Fig. 2). As presented in Fig. 1d, sweeping the frequency of the infrared pump pulse shows that the obtained intensity of ΔOD is linearly proportional to the spectral overlap of the pump and the absorbance of the N-H-*as* modes. Furthermore, an off-resonance pump (centered at 2200 cm^−1^) results in negligible transient response (Supplementary Fig. 3), confirming that the response shown in Fig. 1c is due to vibrational excitation of the organic sublattice. The nanosecond timescale relates slow down-conversion of thermal energy from the high-energy N-H-*as* modes to the low-lying, cold phonon bath, with the latter encompassing all the CH_3_NH_3_^+^ modes except the N-H-*as* modes, and all the modes of the inorganic sublattice.

**Fig. 2:**
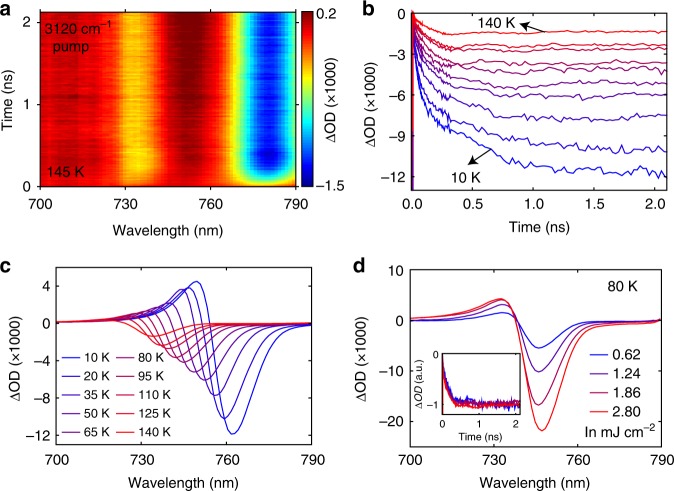
Temperature- and fluence-dependent transient optical response. **a** ΔOD spectral map measured at 145 K with on-resonance infrared pump. **b** Transient kinetics at the wavelength that shows the strongest bleach signal (indicated by the dashed line in Fig. 1c), measured at different temperatures. **c** Transient spectra at 2 ns delay time measured at different temperatures. Legend in (**c**) applies to (**b**) as well. Pump fluence used in (**b**, **c**) was 0.45 mJ cm^−2^. **d** Fluence-dependent transient optical spectra at 2 ns delay time; inset: kinetics for different fluences appear nearly unchanged

Probing the tetragonal phase at 205 K distills a comparably faster thermal equilibration with a timescale of hundreds of picoseconds (Supplementary Fig. 4). Measurement near the phase transition (near 145 K) reveals that the two coexisting phases respond independently to the pump (Fig. 2a), as no evidence of transient phase change is observed, which would otherwise lead to the transfer of spectral weight between the two phases. The ΔOD amplitude near the bandgap obtained for the tetragonal phase is, as expected, weaker than that for the orthorhombic counterpart, owing to a weaker vibrational absorption (Fig. 1b). The nearly identical timescales of ΔOD kinetics for the coexisting phases at 145 K (Supplementary Fig. 4d) indicate that the rotations of CH_3_NH_3_^+^, which are only present in the tetragonal phase, do not apparently influence the phonon–phonon coupling strength between the two sublattices. Temperature-dependent kinetics (Fig. 2b) reveal slower thermal equilibration at lower temperatures. Furthermore, the ΔOD amplitude increases with decreasing temperature (Fig. 2c), which results from the heat capacity that diminishes, and the absorption cross-section of the N-H-*as* modes that grows, with decreasing temperature. Fluence-dependent measurements (Fig. 2d) show that the ΔOD amplitude scales nearly linearly with the pump fluence, and the thermal equilibration time is not sensitive to pump fluence over the explored range, consistent with earlier work based on transient mid-infrared spectroscopy using electronic excitation and an infrared probe^31^.

**Fig. 3:**
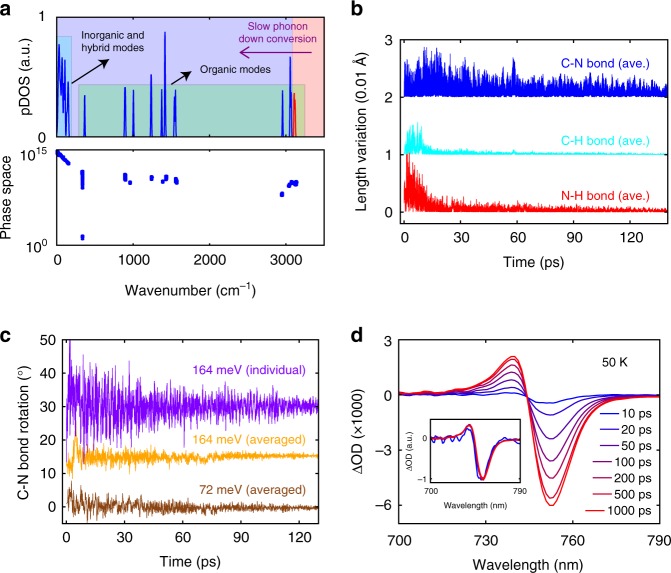
First-principles calculation of the phonon dynamics for orthorhombic CH_3_NH_3_PbI_3_. **a** Calculated zero-Kelvin phonon density of states (pDOS) and phonon scattering phase space. **b** Absolute variation in the N-H (red), C-H (cyan), and C-N (blue) bond lengths from ab initio molecular dynamics (AIMD) simulations. Data are averaged over the four CH_3_NH_3_^+^ cations in the orthorhombic unit cell. Initial imparted energy is 164 meV (per unit cell). **c** Variation in the C-N bond angle for different, indicated amounts of energy imparted into the unit cell. The purple curve is for a representative C-N bond; orange and brown curves are data averaged over all of the four CH_3_NH_3_^+^ cations in the orthorhombic unit cell. Data in (**b**, **c**) are offset for clarity. **d** Transient ΔOD spectra acquired at several different delay times; inset shows the overlapped transient spectra in arbitrary units by simple scaling. Fluence used was 0.45 mJ cm^−2^

Having established the transient electronic response around the bandgap following infrared molecular vibrational excitation, we then turn to investigating whether perturbatively induced motions of the organic sublattice influence the near-bandgap optical properties of CH_3_NH_3_PbI_3_. As illustrated in Fig. 3a, following the selective excitation of N-H-*as* modes, phonon–phonon scattering leads to the sequential population of various lower-energy CH_3_NH_3_^+^ vibrational modes, and ultimately the population of inorganic phonon modes. Note that thermal equilibration within the inorganic sublattice itself requires only 10 to 20 ps, as shown by ultrafast electron diffraction measurements^4^. Therefore, the nanosecond timescale is mainly attributed to the following two consecutive processes: (i) intramolecular vibrational relaxation (IVR) within the CH_3_NH_3_^+^ cations^34^, and (ii) non-equilibrium thermal energy transfer from CH_3_NH_3_^+^ to the inorganic framework^31^. The IVR process of the CH_3_NH_3_^+^ cations involves a succession of energy down-conversion through all the intermediate phonon modes starting from the selectively excited organic mode to the lowest-lying modes, which is expected to take longer than the lifetime of any specific organic vibrational mode. Indeed, several two-dimensional infrared spectroscopic studies demonstrated a few-picosecond timescale for the reorientational motions of the organic cations, as well as a significantly longer timescale (tens to hundreds of picoseconds) that was attributed to thermal response of the lattice^13–15^. Although the longer timescale has not been further pursed in those studies, we speculate that it is of similar origin as what we observed here. We note that the slow timescale can also be contributed by an ineffective (i.e., slow) heat energy transfer from the organic vibrational modes to the inorganic phonon modes, evident from the low thermal conductivities of organic–inorganic hybrid material systems^35,36^. We also note that the coupling between the high-energy vibrations and the electronic states during the first 10 ps cannot be carefully addressed due to the excitation of optical phonon modes of the lead iodide framework (as discussed in the Time-resolved photoluminescence measurements section).

To gain more insights into the vibrational evolution, we performed ab initio molecular dynamics simulations on the orthorhombic structure with selective excitation of the N-H-*as* modes based on the phonon calculations, from which we extracted atomic trajectories up to 130 ps. As shown in Fig. 3b, selective excitation of the N-H-*as* modes results in the population of various stretching modes as represented by the variation of bond lengths of N-H, C-H, and C-N. The C-N bond-length variation decays more slowly in comparison to the C-H and N-H bonds, as the latter two are associated with vibrational modes of the highest frequencies and depopulate most rapidly via IVR. Note that librational motions, captured by the orientational variation of the C-N bond (Fig. 3c), can also be induced. The simulated timescale is furthermore not strongly dependent on the total imparted energy in the simulations (Fig. 3c), which is consistent with the experimental results (Fig. 2d).

 The change of the ΔOD amplitude can act as a proxy for how much thermal energy has been transferred to the inorganic framework (that in turn determines the bandgap). The transient response at long delay time (later than 1000 ps), when the sample has reached thermal equilibrium (Fig. 2b), results from the change of the bandgap with temperature. Transient spectra plotted for representative delay times (Fig. 3d) during the thermal relaxation closely overlap with a simple scaling of the amplitude. Such spectral similarity suggests that no net increase or decrease of optical absorption coefficient, other than that caused by lattice heating, occurs upon this perturbation when CH_3_NH_3_PbI_3_ is at thermal non-equilibrium (i.e., 0 to 1000 ps). Because optical absorption can be viewed as an inverse process of bimolecular recombination^37^, the data shown in Fig. 3d implicitly suggest that the pump-induced CH_3_NH_3_^+^ motions do not impact bimolecular recombination in CH_3_NH_3_PbI_3_.

IPEP measurements on CH_3_NH_3_PbBr_3_ and CH_3_NH_3_PbCl_3_ single crystals reveal similar transient optical response (Supplementary Fig. 5) compared to CH_3_NH_3_PbI_3_ due to their structural similarity. However, in contrast to CH_3_NH_3_PbX_3_ (X = I, Br, Cl), which are three-dimensional (3D) perovskites, the impact of artificially induced organic cation motion is observed for the two-dimensional perovskite, (CH_3_(CH_2_)_3_NH_3_)_2_PbI_4_. For this compound^38^, the CH_3_(CH_2_)_3_NH_3_^+^ cations situated between the perovskite layers strongly impact the electronic properties through both quantum and dielectric confinement effects. Following selective vibrational excitation near 3100 cm^−1^ (Supplementary Fig. 6), a much faster transient optical response, relative to the 3D counterparts, was observed for (CH_3_(CH_2_)_3_NH_3_)_2_PbI_4_ near the exciton transition energy (Supplementary Fig. 5), owing to the internal IVR processes of the CH_3_(CH_2_)_3_NH_3_^+^ cations that alter the strength of quantum confinement of the electron–hole pairs (i.e., excitons) residing on the Pb-I framework.

### Time-resolved photoluminescence measurements

**Fig. 4:**
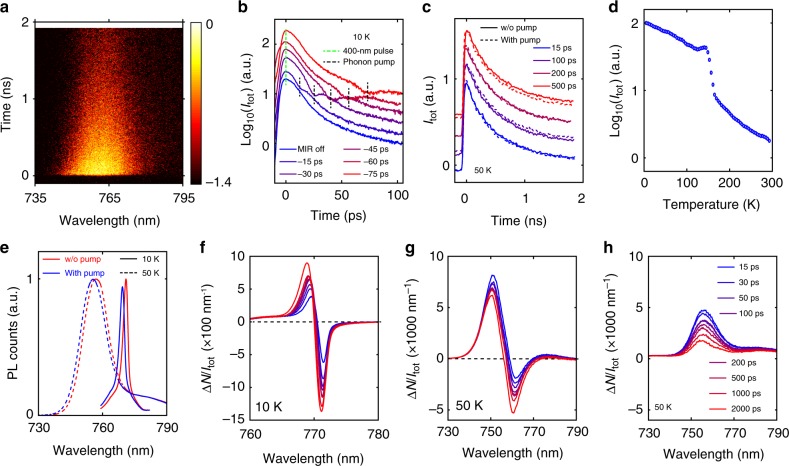
Infrared pump photoluminescence (PL)-probe measurements of CH_3_NH_3_PbI_3_. **a** Spectrally and temporally resolved PL counts measured with a streak camera (scalebar is in log_10_ scale) without using the infrared pump. **b** Decay kinetics of the PL intensity acquired under various negative Δ*t* values. **c** Decay kinetics of the PL intensity acquired under various positive Δ*t* values, with (dashed lines) and without (solid lines) using the infrared pump. **d** Static, temperature-dependent, wavelength-integrated PL intensity of the CH_3_NH_3_PbI_3_ film. **e** Time-integrated PL spectra acquired at 10 K and at 50 K, with Δ*t* = 2000 ps. Relative change of the time-integrated PL spectra with various positive delay times measured at 10 K in (**f**), and measured at 50 K in (**g**, **h**). An on-resonance (3120 cm^−1^) infrared pump was used in (**e**, **f**, **g**); an off-resonance (2200 cm^−1^) infrared pump was used in (**h**). Fluence of the infrared pump was fixed at 3.8 mJ cm^−2^ in these measurements. Legend in (**h**) also applies to (**f**, **g**)

We further explored if the infrared pump-induced CH_3_NH_3_^+^ motions can directly impact photoluminescence (PL) of CH_3_NH_3_PbI_3_, using infrared pump PL-probe measurements based on the schematic shown in Supplementary Fig. 7. Here, the pump pulse was followed by a time-delayed (denoted as Δ*t*), low-intensity, 400 nm PL-probe pulse that generates electron–hole pairs (positive Δ*t* is defined if the infrared pump hits the sample first). The emitted photons were either detected by a charge-coupled device (CCD) camera or spectrally and temporally resolved by a streak camera. Figure 4a presents a representative, spectrally resolved PL map measured at 50 K without using the infrared pump. Figure 4b shows the spectrally integrated PL decay kinetics measured under various negative values of Δ*t*, with the infrared pump turned on. The dip in the PL decay trace, which exhibits an instrument-response-time-limited timescale, is also observed by using an off-resonant mid-infrared pump (Supplementary Fig. 8), and therefore attributed to the electric-field-driven dissociation of electron–hole pairs, or the optical Stark effect. We then focused on measurements under positive Δ*t* values. Figure 4c presents PL decays measured using different positive Δ*t* values that traverse the duration of sublattice thermal equilibration. We find that optically pumping the organic vibrational modes of CH_3_NH_3_PbI_3_ does not apparently alter the carrier recombination rate.

While streak camera measurements only detect PL over specific time windows, time-integrated PL measurements with higher signal-to-noise ratio can reveal whether the infrared pump-induced CH_3_NH_3_^+^ motions can enhance (by suppressing defect scattering of charge carriers) or suppress (by slowing down the recombination rate of carriers so that more trapping events can take place) the brightness of CH_3_NH_3_PbI_3_. Static, temperature-dependent PL intensity (Fig. 4d) suggests that a substantial portion of photo-generated carriers are lost through non-radiative decay pathways at 50 K. The time-integrated PL spectra measured at 10 K and 50 K are shown in Fig. 4e; note that the dramatic change in PL linewidth upon temperature reduction has been observed previously^39^. For both temperatures, the infrared pump (Δ*t* = 2000 ps) yields a blueshift of the PL spectrum, stemming from a bandgap increase due to lattice heating. We ran measurements at these two temperatures because the fast PL decay component due to bimolecular recombination^40^ is commensurate with the thermal equilibration time, and hence the impact of non-equilibrium CH_3_NH_3_^+^ motions, if there are any, can be more pronounced. Based on the PL spectral shift at 50 K (in conjunction with data in Fig. 1a and heat capacity^41^), we estimate that 0.06 photons are absorbed per orthorhombic unit cell by CH_3_NH_3_^+^, with a photon energy of 386 meV (corresponding to 3120 cm^−1^). We denote the change of PL counts at each wavelength as Δ*N* = *N*_on_ − *N*_off_, where *N*_on_ (*N*_off_) designates PL counts obtained with (without) the infrared pump. The spectra of Δ*N*/*I*_tot_ measured at 10 K and 50 K using variable positive Δ*t* values are presented in Fig. 4f, g, respectively, where *I*_tot_ is the spectrally integrated, total PL counts measured without the infrared pump. At 10 K, infrared pumping yields a PL blueshift, the magnitude of which increases with Δ*t*. This arises because a larger Δ*t* results in a more substantial sample heating (which causes a larger bandgap increase) before the photoluminescence event takes place. A similar PL blueshift is observed also at 50 K, although an additional increase of the PL intensity, implied by an asymmetric line shape, is observed especially for small Δ*t* values. To explore the origin of such PL enhancement, we ran control experiments with an off-resonance infrared pump centered at 2200 cm^−1^. As shown in Fig. 4h, although the PL blueshift is no longer present due to the lack of lattice heating, a PL intensity enhancement is still recovered. The observed PL enhancement retained using both pumping wavelengths is likely attributable to polaron formation arising from below-bandgap excitation of various phonon modes of the inorganic sublattice (evident from Supplementary Fig. 9)^6^ that may contribute to the protection of charge carriers from defect scattering. This effect is not clearly observable at 10 K since the Δ*N*/*I*_tot_ intensity arising from bandgap blueshift is an order of magnitude larger than in the 50 K measurement. Importantly, comparison of Fig. 4g, h reveals that the induced CH_3_NH_3_^+^ motions alone lead to negligible variation of the integrated PL intensity.

## Discussion

Our transient absorption and PL measurements both suggest that infrared pump-induced CH_3_NH_3_^+^ motions do not lead to noticeable changes of the near-bandgap absorption and PL properties, beyond effects due to lattice heating. Such an observation implies that photo-generated carriers residing on the inorganic sublattice are essentially decoupled from the dynamically disordered organic cations. The transient spectroscopic technique reported here, complementary to fully electronic pump–probe, or fully infrared (vibrational) pump–probe measurements^14,42,43^, can be generalized for studying organic semiconductors, organic–inorganic interfaces, and other organic–inorganic hybrid materials such as superatomic solids^35^ and two-dimensional hybrid perovskites^44^.

## Methods

### Chemicals and the synthesis of CH_3_NH_3_I

Methylamine solution (CH_3_NH_2_, 40 wt.% in H_2_O), *N*-methyl-2-pyrrolidinone (NMP), and γ-Butyrolactone (GBL, ≥99%) were purchased from Aldrich. Hydriodic acid (HI, 57 wt.% in H_2_O) and lead(II) iodide (PbI_2_, 99.9985% metal basis) were purchased from Alfa Aesar. Ethyl ether (anhydrous) and acetone were obtained from Fisher Chemical. All chemicals were used as received without further purification. Equimolar of HI was dropwise added to CH_3_NH_2_ with stirring in a 50 mL round bottom flask immersed in ice bath, followed by rotary evaporation at 60 °C to dry off the solvent. Next, the white solid was obtained and washed with excessive ethyl ether on a filter paper, accompanied with vacuum filtration. The washed CH_3_NH_3_I powder was then dried in a vacuum oven at 60 °C overnight to yield the final product.

### CH_3_NH_3_PbI_3_ thin film fabrication

The preparation of CH_3_NH_3_PbI_3_ thin film follows method reported previously with slight modification^45^. The 1.2:1 molar ratio of CH_3_NH_3_I/PbI_2_ was dissolved in 1:1 volume ratio of NMP/GBL to make a 40 wt.% precursor solution. Then, the precursor solution was spun-coated on an acetone-cleaned sapphire substrate with a speed of 3000 rpm for 30 s. Next, the as-formed wet film was quickly transferred into an ethyl ether bath (50 mL) for 90 s. Finally, the developed film was dried in air, covered by a Petri dish, and followed by annealing at 150 °C for 15 min on a hot plate in a humidity-controlled environment.

### Static optical characterization

Static infrared absorbance spectra were captured with Fourier transform infrared (FTIR; Thermo Nicolet 6700). Static visible absorbance spectra were obtained with a customized setup. In all the optical measurements (both static and transient), the samples were mounted in a 4 K closed-cycle cold-finger cryostat under with a base pressure below 1 × 10^−7^ Torr.

### Transient absorption measurements

Transient absorption (i.e., IPEP) measurements were performed using a 35 fs amplified titanium/sapphire laser operating at 800 nm at a repetition rate of 2 kHz. Broadband visible probe pulses were generated by focusing a portion of the amplifier output onto an Al_2_O_3_ window. Infrared pump pulses were generated by difference frequency mixing of signal and idler beams using an optical parametric amplifier and were reduced in repetition rate to 1 kHz. The probe pulses were mechanically time delayed using a translation stage and retroreflector. Full transient spectral maps are shown in Supplementary Figs. 10 and Fig. 11.

### Infrared pump PL-probe measurements

Generation of the infrared pump pulse was identical to that employed in the transient absorption measurements. The 400 nm PL excitation pulses were produced by frequency-doubling of the 800 nm amplifier output with a BBO crystal and underwent similar beam path as the visible probe pulses in the IPEP measurements. Both the infrared pump pulses and the 400 nm PL excitation pulses were maintained at 2 kHz. Temporally and spectrally resolved PL data were collected with a streak camera. Time-integrated PL data were captured with a CCD camera. The spot size of the 400-nm PL excitation pulses was adjusted to be smaller than that of the infrared pump pulses. The fluence of 400 nm PL excitation pulse was kept below 1 µJ cm^−2^.

### Calculations of the phonon spectrum and phonon–phonon scattering phase space

Vienna Ab initio Simulation Package (VASP) was used to perform structure relaxation and self-consistent density functional theory (DFT) calculations^46–49^. The projector augmented wave (PAW) method^50^ was used in conjunction with the generalized gradient approximation (GGA)^51^ for the exchange-correlation (xc) functional^52^. We used the van der Waals density functional (vdW-DF) method to treat the dispersion forces from non-local electron–electron interactions, particularly, the optB86b-vdW functional^53^. DFT calculations of primitive cell of CH_3_NH_3_PbI_3_ in the orthorhombic phase with space group of *Pnma* (No. 62) were performed using a 4 × 4 × 4 Γ-centered **k**-point mesh and a kinetic energy cutoff of 550 eV. The force and energy convergence thresholds were set to be 5 × 10^−3^ eV Å^−1^ and 10^−8^ eV, respectively. Phonon calculations were performed using Phonopy package^54^, wherein 1 × 1 × 1 supercell structures were constructed to extract harmonic interatomic force constants which were used to construct dynamical matrix and obtain phonon dispersion relation along high-symmetry paths in the Brillouin zone. Phonon scattering phase space accounting for three-phonon interactions was calculated using ShengBTE package^55^.

### Ab initio molecular dynamics (AIMD) simulations

The starting structures for AIMD were obtained by exciting all the CH_3_NH_3_^+^ cations by a certain fraction of the phonon eigenvectors for the highest energy mode (the N-H-*as* mode). To study the effect of fluence on the perovskite dynamics, the atoms in the CH_3_NH_3_^+^ cations were displaced from their equilibrium positions by a fraction of 0.10 and 0.07, which correspond to an energy imparted of 164 meV and 72 meV, respectively. AIMD simulations were performed as implemented in the VASP package, using PAW pseudopotentials and the GGA functional with vdW-DF correction. Because of computational expense, spin–orbit coupling was not considered. An energy cutoff of 500 eV was used for the plane wave basis. The atoms were relaxed until forces on the atoms were less than 0.0001 eV Å^−1^, and gamma point sampling was used for the Brillouin zone. A time step of 0.5 fs was applied for the AIMD calculations. Standard molecular dynamics was performed using a microcanonical ensemble by applying the IBRION = 0 and the SMASS = −3 tags. The AIMD trajectories and structure snapshots were visualized with VESTA and VMD softwares^56,57^. The structure of the CH_3_NH_3_PbI_3_ used in the calculation was obtained from ref. ^58^.

## Supplementary information

Supplementary Information

## Data Availability

All relevant data used in the article are available from the authors.
