# Supplementary material for: Infrared-pump electronic-probe of methylammonium lead iodide reveals electronically decoupled organic and inorganic sublattices
Source: Nat Commun. 2019 Jan 29;10:482. doi: 10.1038/s41467-019-08363-2 (PMC6351559; doi:10.1038/s41467-019-08363-2)
Supplement: Supplementary file 1 — Supplementary Information [file 41467_2019_8363_MOESM1_ESM.pdf]

Infrared-pump electronic-probe of methylammonium lead iodide reveals electronically decoupled organic and inorganic sublattices

P. Guo et al.

Supplementary Information for

Infrared-pump electronic-probe of methylammonium lead iodide reveals electronically decoupled organic and inorganic sublattices

Peijun Guo[1], Arun Mannodi-Kanakkithodi[1], Jue Gong[2], Yi Xia[1], Constantinos C. Stoumpos[3], Duyen H. Cao[4], Benjamin T. Diroll[1], John B. Ketterson[5], Gary P. Wiederrecht[1], Tao Xu[2], Maria K. Y. Chan[1], Mercouri G. Kanatzidis[3], Richard D. Schaller[1,3,*]

[1]Center for Nanoscale Materials, Argonne National Laboratory, 9700 South Cass Avenue, Lemont, IL 60439, United States

[2]Department of Chemistry and Biochemistry, Northern Illinois University, 1425 W. Lincoln Hwy., DeKalb, IL 60115, United States

[3]Department of Chemistry, Northwestern University, 2145 Sheridan Road, Evanston, IL 60208, United States

[4]Materials Science Division, Argonne National Laboratory, 9700 South Cass Avenue, Lemont, IL 60439, United States

[5]Department of Physics and Astronomy, Northwestern University, 2145 Sheridan Road, Evanston, IL 60208, United States

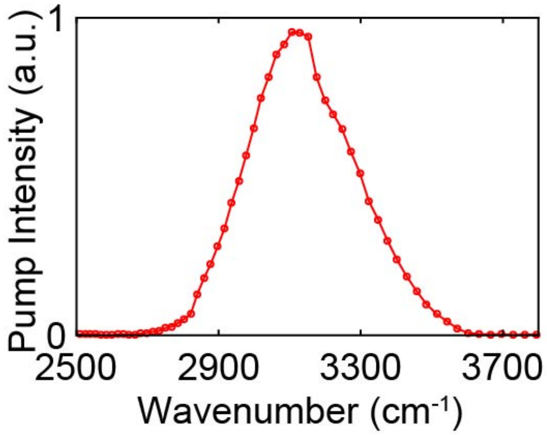

Supplementary Figure 1. Spectral profile of the on-resonance infrared pump used for $MAPbI_3$ (centered at 3120 cm^{-1}).

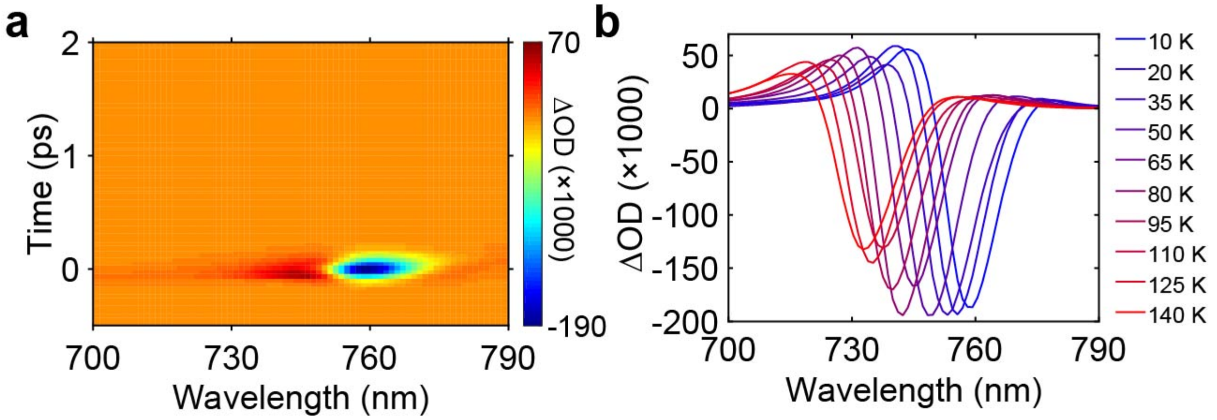

Supplementary Figure 2. Transient response near zero delay time showing significant change of the bandgap resulting from the optical Stark effect. a ΔOD spectral map of $CH_3NH_3PbI_3$ acquired at 10 K. **b** Temperature dependent ΔOD spectra acquired at zero delay time. Fluence used for these measurements was kept at 0.45 mJ·cm^{-2}.

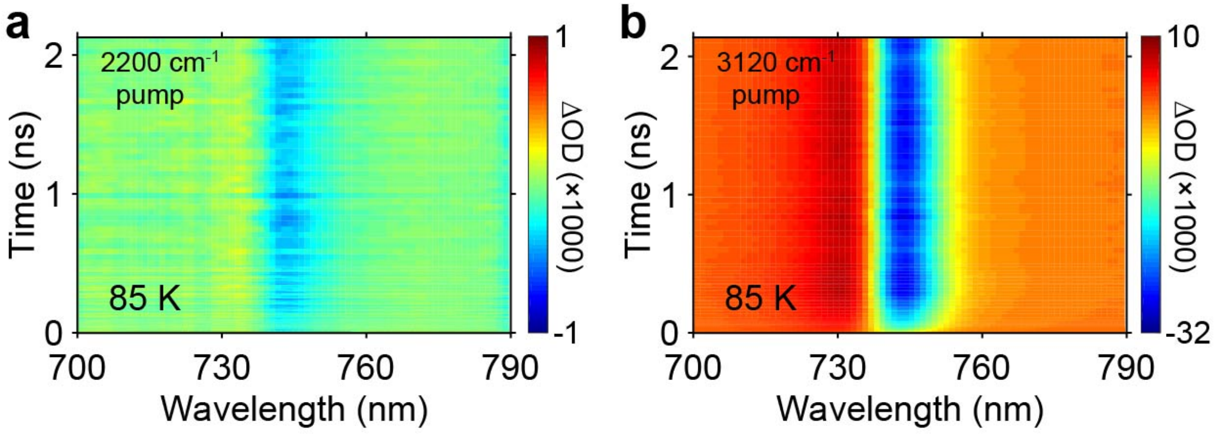

Supplementary Figure 3. Comparison of off-resonance infrared pumping with on-resonance infrared pumping. a ΔOD spectral map with off-resonance infrared pump (centered at 2200 cm^{-1}) measured at 85 K. **b** ΔOD spectral map with on-resonance infrared pump (centered at 3120 cm^{-1}) measured at 85 K. The same fluence was used for **a** and **b**. Note that different scalebars are used for the two spectral maps.

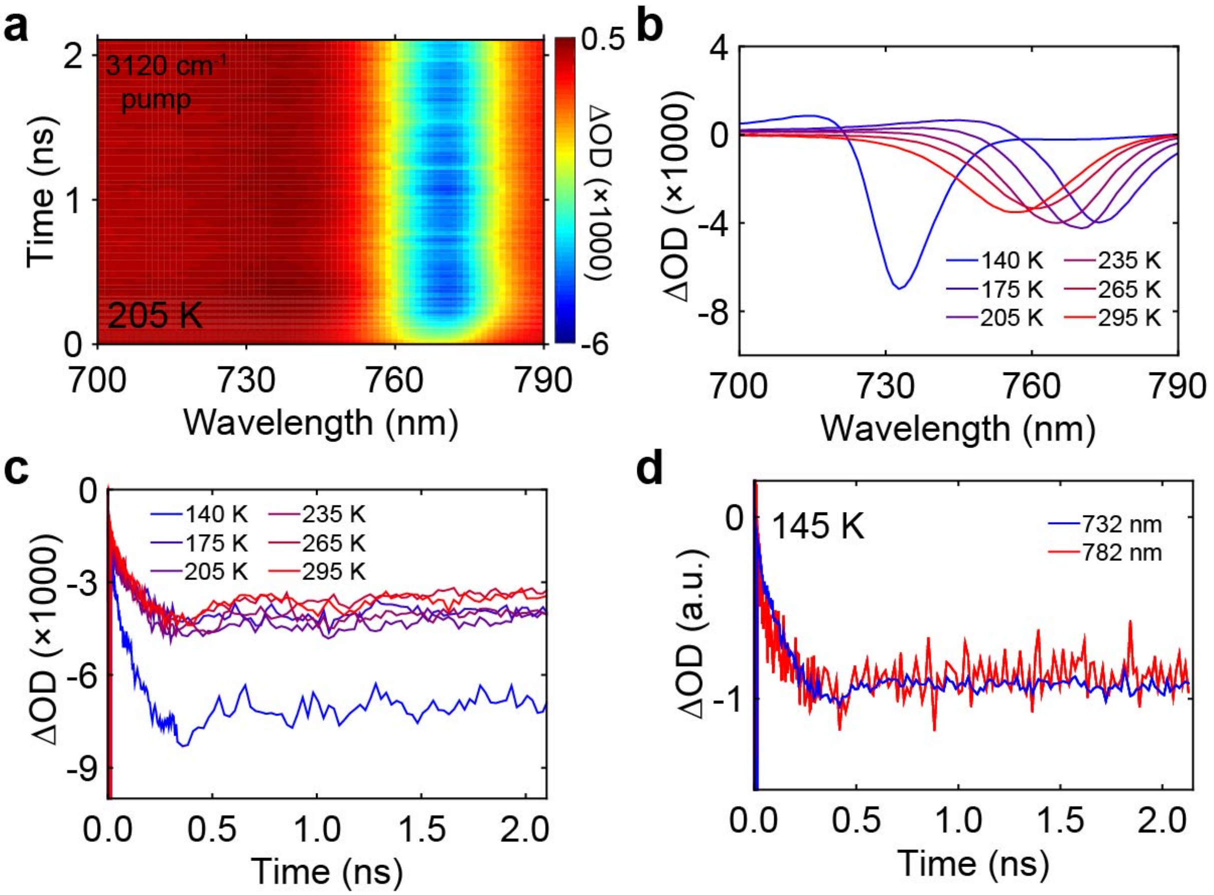

Supplementary Figure 4. Transient spectral response of $CH_3NH_3PbI_3$ in the tetragonal phase measured with on-resonance infrared pump (centered at 3120 cm^{-1}). a ΔOD spectral map measured at 205 K. **b** ΔOD spectra at 2-ns delay time measured at different temperatures. **c** ΔOD kinetics extracted at the wavelength of the negative ΔOD peak measured at different temperatures. **d** Comparison of the ΔOD kinetics at 732 nm and 782 nm (extracted from data shown in Fig. 1d in the main text).

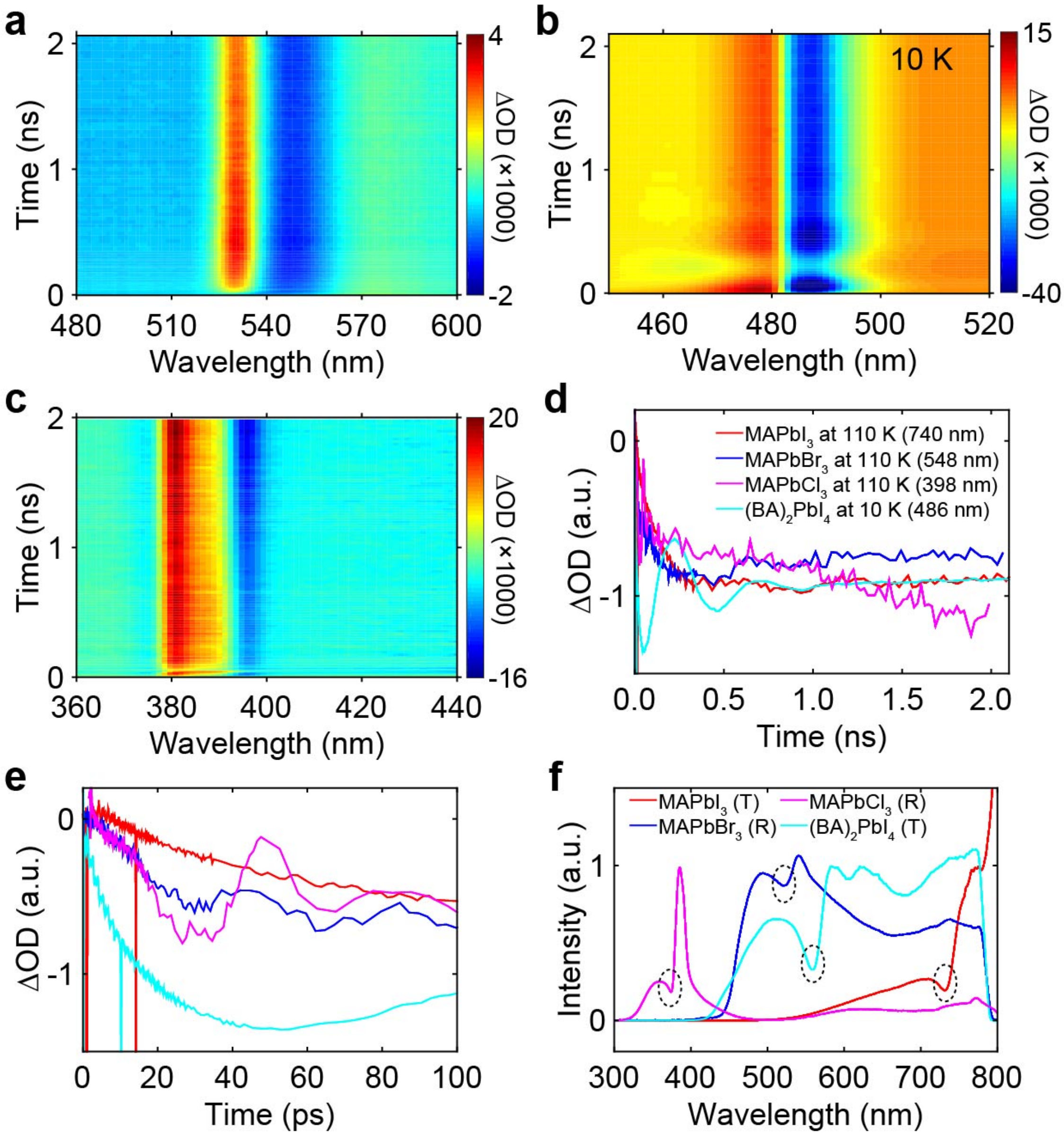

Supplementary Figure 5. **a** ΔOD spectral map measured at 110 K for $CH_3NH_3PbBr_3$ single crystal in the reflection geometry. **b** ΔOD spectral map measured at 10 K for layered perovskite $(CH_3(CH_2)_3NH_3)_2PbI_4$ thin film in the transmission geometry. **c** ΔOD spectral map measured at 110 K for $CH_3NH_3PbCl_3$ single crystal in the reflection geometry. **a**, **b** and **c** were obtained with on-resonance infrared pumping. **d** Comparison of the ΔOD kinetics (in arbitrary units) at selected wavelengths. **e** Same as **d** but plotted for the time window of 0~100 ps. **f** Probe spectra obtained for the samples in the transmission (T) or reflection (R) geometry; the dashed circles highlight the exciton features. The synthesis of $CH_3NH_3PbBr_3$, $CH_3NH_3PbCl_3$ and $(CH_3(CH_2)_3NH_3)_2PbI_4$ single crystals followed literature-reported procedures[1, 2, 3]. The oscillations of the transient signals observed in **d** and **e** are due to acoustic phonons of the $CH_3NH_3PbCl_3$ single crystal or breathing mode of the $(CH_3(CH_2)_3NH_3)_2PbI_4$ thin film[4].

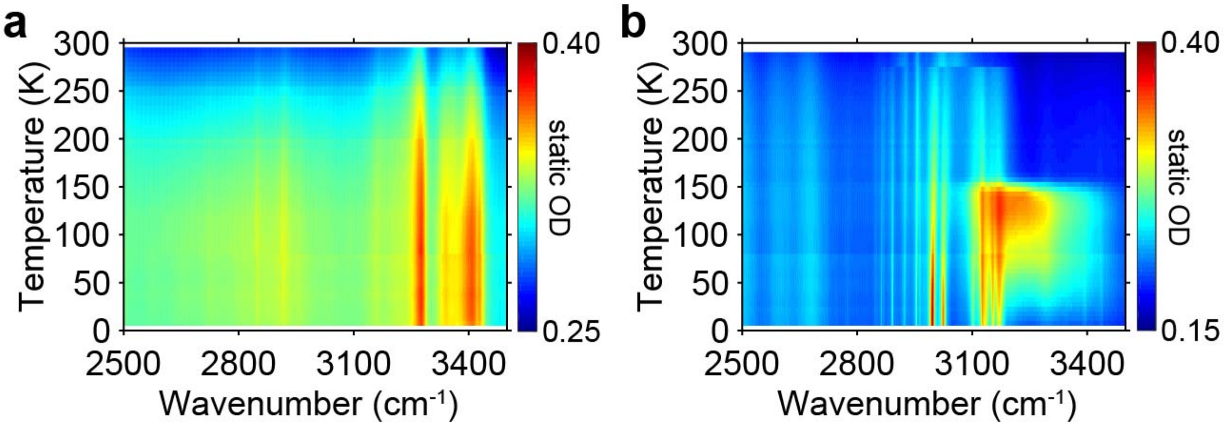

Supplementary Figure 6. Static, temperature dependent absorbance of the vibrational modes in the range of 2500 cm^{-1} to 3500 cm^{-1} for $MAPbBr_3$ thin film in **a** and for $(CH_3(CH_2)_3NH_3)_2PbI_4$ thin film in **b**.

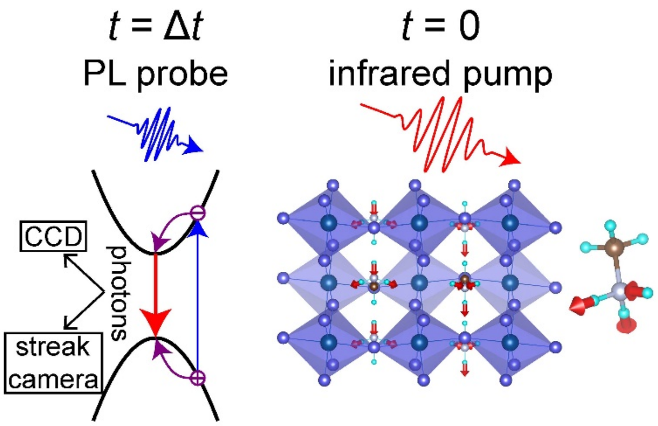

Supplementary Figure 7. Schematic illustration of the infrared-pump photoluminescence (PL)-probe experiment. The crystal structure was taken from reference[5].

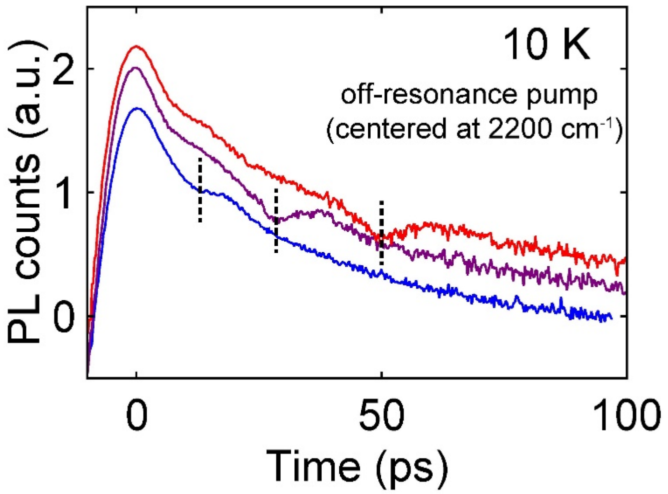

Supplementary Figure 8. PL intensity decay kinetics acquired under various negative Δt values; an off-resonance (2200 cm^{-1}) infrared pump was employed after excitation of the sample at 400 nm.

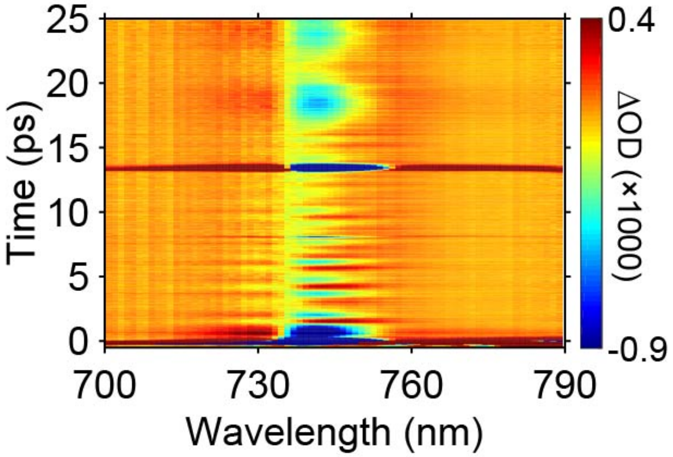

Supplementary Figure 9. Transient ΔOD spectral map measured at 10 K with off-resonance infrared pump centered at 2200 cm^{-1}. The oscillations of ΔOD near the bandgap indicate the excitation of optical phonon modes in the inorganic sublattice[6].

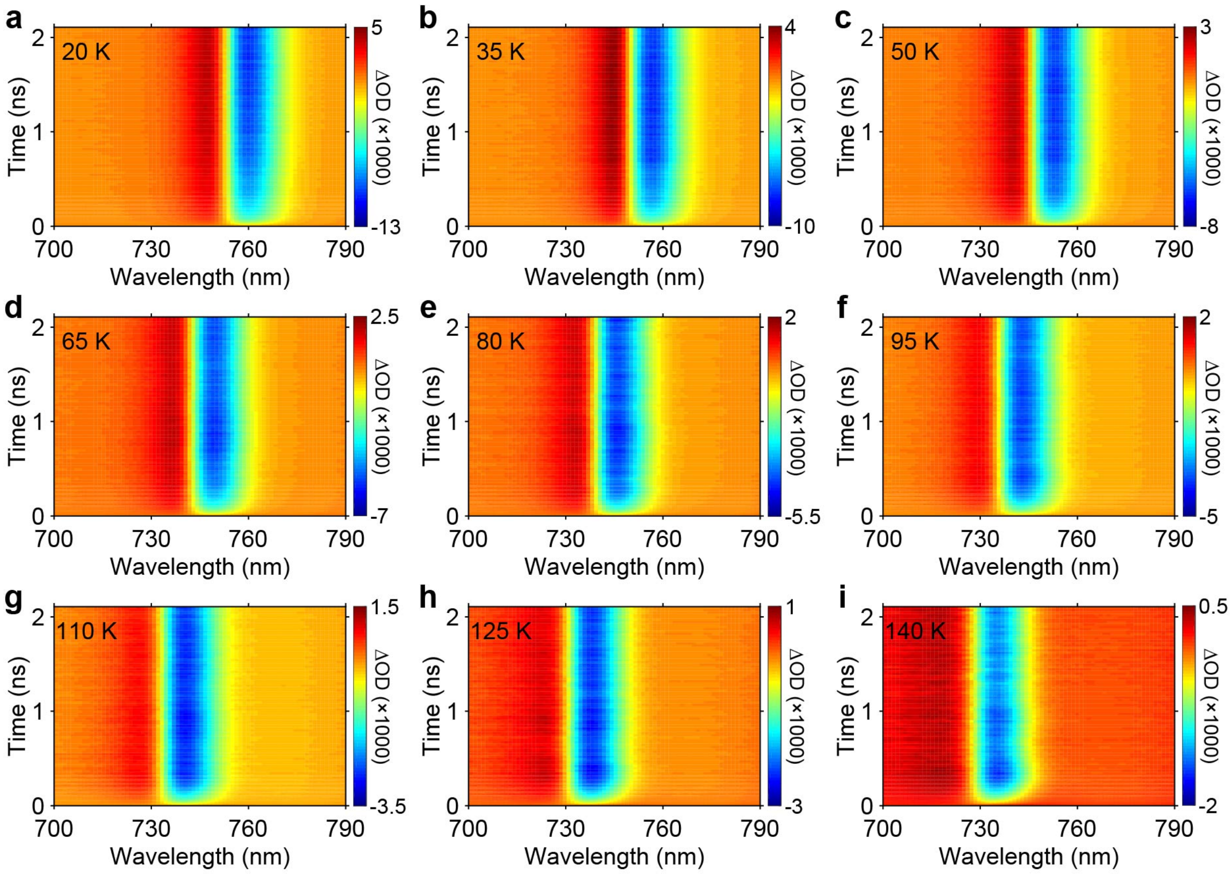

Supplementary Figure 10. Full transient ΔOD spectral maps measured at different temperatures with on-resonance infrared pump (centered at 3120 cm^{-1}, fluence was 0.45 $mJ \cdot cm^{-2}$).

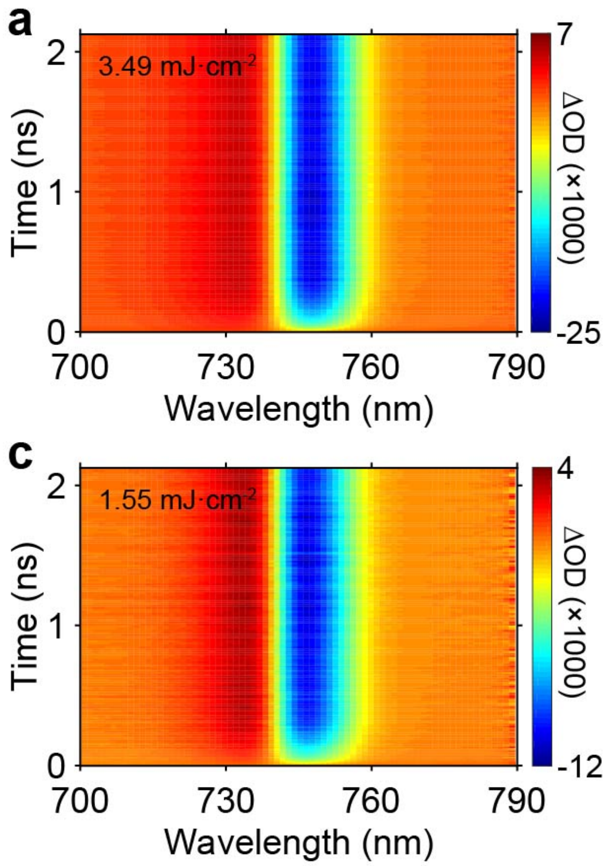

Supplementary Figure 11. Full transient ΔOD spectral maps measured at 80 K under different fluences with on-resonance infrared pump (centered at 3120 cm^{-1}).

Supplementary References

1. Saidaminov, M.I. et al. High-quality bulk hybrid perovskite single crystals within minutes by inverse temperature crystallization. *Nat. Commun.* **6**, 7586 (2015).
2. Stoumpos, C.C. et al. Ruddlesden–Popper hybrid lead iodide perovskite 2D homologous semiconductors. *Chem. Mater.* **28**, 2852-2867 (2016).
3. Maculan, G. et al. $CH_3NH_3PbCl_3$ single crystals: inverse temperature crystallization and visible-blind UV-photodetector. *J. Phys. Chem. Lett.* **6**, 3781-3786 (2015).
4. Guo, P. et al. Cross-plane coherent acoustic phonons in two-dimensional organic-inorganic hybrid perovskites. *Nat. Commun.* **9**, 2019 (2018).
5. Brivio, F. et al, Lattice dynamics and vibrational spectra of the orthorhombic, tetragonal, and cubic phases of methylammonium lead iodide. *Phys. Rev. B* **92**, 144308 (2015).
6. Wang, H., Valkunas, L., Cao, T., Whittaker-Brooks, L. & Fleming, G.R. Coulomb screening and coherent phonon in methylammonium lead iodide perovskites. *J. Phys. Chem. Lett.* **7**, 3284-3289 (2016).
